# Comparison of silicon oil removal with various viscosities after complex retinal detachment surgery

**DOI:** 10.1186/1471-2415-6-21

**Published:** 2006-05-31

**Authors:** Masoud Soheilian, Mohammad Mazareei, Mehrdad Mohammadpour, Bahram Rahmani

**Affiliations:** 1 Ophthalmology Department and Ophthalmic Research Center Labbafinejad Medical Center, Shaheed Beheshti University of Medical Sciences, Tehran, 16666 –, Iran

## Abstract

**Background:**

Despite the progress in vitreoretinal surgery and the importance of silicone oil as an adjunct for the treatment of complex forms of retinal detachment, controversy still surrounds the issue of selecting the proper oil viscosity for clinical use. Herein, we evaluate the outcomes of retinal detachment (RD) surgery after removing silicone oils of different viscosities.

**Methods:**

In this retropsective cohort study, eighty-two eyes with surgically re-attached retinas, of which 53 were filled with 5000cs silicone oil and 29 with 1000cs silicone oil were enrolled. We evaluated the outcomes and complications following silicone oil removal. Final anatomic success (stable re-attachment), final visual acuity (VA) and intraocular pressure (IOP)were recorded and analysed.

**Results:**

Of 82 eyes, 41 had proliferative vitreoretinopathy (PVR), 24 were associated with intraocular foreign bodies, 10 had endophthalmitis and 7 had proliferative diabetic retinopathy with tractional retinal detachment. Prior to silicone oil removal, the retina was attached in all eyes, 29% had VA ≥ 6/120 and 52% had IOP ≥ 21 mmHg. After silicone oil removal, the retina remained attached in 59(72%) of the eyes, 34% had VA ≥ 6/120 and 9% had IOP ≥ 21 mmHg. Comparing 1000cs and 5000cs silicone oil filled eyes, redetachment occurred more frequently in the latter group especially in cases with associated PVR. Final VA worse than 6/120 was associated with initial VA < 6/120 (OR = 32.2 95%CI 7.4–140.2) and use of 5000cs silicone oil (OR = 7.9 95%CI 1.9–32.2). No factor was significantly associated with final IOP ≥ 21 mmHg.

**Conclusion:**

In complicated retinal detachment surgery, use of 5000cs silicone oil may be associated with a poorer anatomic and visual outcome compared with 1000cs silicone oil. However there was no difference between the two viscosities in IOP elevation. A randomized controlled study is necessary to further evaluate such a possibility.

## Background

Despite the progress in vitreoretinal surgery and the importance of silicone oil as an adjunct for the treatment of complex forms of retinal detachment, controversy still surrounds the issue of selecting the proper oil viscosity for clinical use[1,2]. There is no difference in the tamponading force effected by silicone oils of different viscosities. The tamponading force relates to interfacial surface tension between the oil and the surrounding tissue [3]. Furthermore, new experiments have disclosed that the optical transmittance, penetration depth and the absorption spectra of mid-infrared cutting lasers through silicone oils of different viscosities are almost identical [4]. However, different studies have shown that lower viscosity silicone oil is prone to earlier emulsification. Therefore, it is a common assumption that side effects such as cataract, glaucoma and keratopathy may increase with the use of lower viscosity oils [2,5,6].

The tamponading force of silicone oil continues until emulsification occurs, therefore, to reduce the side effects it is important to use the most suitable viscosity of silicone oil and to remove it once its function for achieving stable reattachment of the retina has been accomplished. In this study, we analyzed and compared the visual outcome, redetachment rate, and complications of two currently available silicone oils with different viscosities (1000cs & 5000cs) after removal in eyes with a stable appearing retina following vitreoretinal procedures for detachment.

## Methods

This study includes 82 eyes of 82 consecutive patients who underwent silicone oil removal from 1986–1997 at a tertiary referral eye center in Tehran. The study was performed in compliance with the Helsinki Declaration after the approval by institutional review board of Labbafinejad Medical Center . Selection of silicone oil viscosity was mostly based on the availability of the oil in the operating room rather than surgeons' choice: throughout the study period, (prior to FDA approval), Labbafinejad Medical Center carried only one silicone viscosity (1000cs or 5000cs) at a given time. Hence, there was no selection bias in terms of the viscosity selected for each case. Before silicone oil removal, all patients had developed different degrees of emulsification, from an early appearance of fish eggs in the superior retina visualized only by indirect ophthalmoscopy, to overt droplets of oil which could be seen in the anterior chamber by slit lamp. All eyes had completely stable, reattached retina prior to silicone oil removal. Oil removal was as complete as possible. All eyes were aphakic and the best-corrected visual acuity was based on performance with a new postoperative correction. Indications for silicone oil removal were any of the following alone or in combination: oil emulsification, high intraocular pressure and keratopathy.

Twenty-nine eyes had been filled with 1000 centistoke silicone oil and 53 with 5000cs silicone oil. The volume of injected oil ranged from 3.5cc to a maximum of 4.5cc. A single surgeon performed the operations on every patient, including all vitreoretinal procedures and subsequent silicone oil removals.

Patients had a complete ophthalmologic examination including refraction and best corrected visual acuity (if possible), slit lamp examination, applanation tonometry, gonioscopy and indirect ophthalmoscopy both prior to and after silicone oil removal. Patient charts were also reviewed for the pre-silicone oil status of the retina including the extent of retinal detachment, macular involvement, grading of PVR, cause of endophthalmitis, location, number and type of retinal breaks in traumatic eye injuries and presence and location of neovascularization in PDR cases. All cases were carefully followed for development of possible complications and maitenance of retinal stability.

### Surgical technique

Since all patients were aphakic, after a localized peritomy, a small superior limbal incision was made and kept open with an iris spatula. An infusion cannula connected to an infusion bottle was placed through the inferior temporal pars plana into the midvitreous cavity. Silicone is light and floats on water, thereby, by allowing the infusion fluid to flow inside the eye, silicone oil was expelled through the superior limbal incision. No other membrane was removed. However, small intraocular silicone bubbles remained postoperatively in all cases. The sclerotomy and the limbal incision were then closed and the conjunctiva was re-approximated. At the conclusion, a subconjunctival injection of antibiotic and steroid was performed.

### Statistical methods

Statistical analysis of patients' data was performed by SAS statistical software. The data was analyzed using chi-square, Fisher's exact tests, Student t-test and multiple logistic regression. Main outcome measures included final visual acuity, redetachment rate and final intraocular pressure following silicone oil removal.

## Results

Eighty-two eyes of 82 patients were studied. Forty-four of the operated eyes (53.6%) were right and 38 (46.4%) were left. Fifty-two of the patients were male (63.4%) and 30 were female (36.6%). All patients were caucasians. Based on surgical indications for silicone oil injection, patients were categorized into 4 groups: complex retinal detachment (RD) associated with PVR (Forty-one eyes), RD associated with intraocular foreign body (IOFB) (24 eyes), RD associated with endophthalmitis (10 eyes) and RD due to complications of proliferative diabetic retinopathy (7 eyes). Pre-silicone oil status of the studied eyes was as follows.

In eyes treated for retinal detachment associated with PVR (41 eyes), the macula was detached in all cases and the extent of retinal detachment ranged from three to four quadrants. PVR grade C (anterior or posterior) was observed in all these cases. Anterior PVR was present in all eyes and involved 12 clock hours in 15 eyes. Posterior PVR was present in 30 eyes involving 6 to 12 clock hours.

In eyes with retinal detachment associated with IOFB (24 eyes), PVR was present in eight eyes preoperatively. Six eyes had multiple large peripheral breaks and three eyes developed giant retinal dialyses during foreign body removal. Seven eyes developed posterior retinal breaks while deeply embedded intraretinal foreign bodies were being dislodged.

In the endophthalmitis group (10 eyes) there were 6 cases of post-traumatic bacterial endophthalmitis; 2 acute postoperative bacterial infections and 2 cases of bleb- associated endophthalmitis. Coagulase-negative Staphylococci accounted for two cases, Streptococci, Haemophilus Influenzae and Bacillus Cereus were isolated each in one case, the rest (5 eyes) were culture-negative.

In the group with complications of proliferative diabetic retinopathy (seven eyes), all had tractional retinal detachment involving the macula and all had been treated with argon laser panretinal photocoagulation. In addition to presence of tractional RD, all eyes displayed different degrees of non-regressed neovascularization of the disc and elsewhere (NVD and NVE).

Each surgical indication was found in approximately equal proportions in the 1000 and 5000 centistoke oil categories [Table 1]. All patients who underwent silicone oil removal had an stable attached retina before the operation for at least 50 days. Indications for silicone oil removal were silicone emulsification alone in 8 eyes (9.7%), emulsification associated with high IOP in 43 eyes (52.4%) and emulsification associated with varying degrees of keratopathy in 31 eyes (37.8%). The mean time to oil removal was 10 ± 6.5 months for eyes filled with 1000cs silicone oil and 23.9 ± 15.1 months for eyes filled with 5000cs silicone oil [table 2]. After silicone oil removal patients were followed from 4 to 120 months. The follow-up period was almost equal for silicone 1000cs and 5000cs filled groups.

**Table 1:** Relation between surgical indication, viscosity of silicone oil and final retinal status

Diagnosis	Type of silicone oil				Final retinal status							
		
	1000 cs		5000 cs		Attached 1000cs 5000cs				Detached 1000cs 5000cs			
	
	No	%	No	%	No	%	No	%	No	%	No	%
RD* + PDR†	5	17	2	4	4	15.3	2	6	1	33.3	0	0
RD + endophthalmitis	4	14	6	11	4	15.3	5	15	0	0	1	5
RD + IOFB‡	8	28	16	30	7	27	10	30.3	1	33.3	6	30
RD + PVR**	12	42	29	55	11	42.3	16	48.5	1	33.3	13	65
Total	29	100	53	100	26	100	33	100	3	100	20	100

*RD = Retinal detachment – †PDR = Proliferative diabetic retinopathy ‡IOFB = Intraocular foreign body – **PVR = Proliferative vitreoretinopathy

**Table 2:** Duration of silicone oil retention before its removal

	Silicone 1000cs		Silicone 5000cs	
	
	No	%	No	%
< 2 months	15	51.7	9	17
2–6 months	13	44.8	24	45.3
6–12 months	1	3.4	18	34
> 12 months	0	0	2	3.7
Total	29	100	53	100

### Anatomic status of the retina following silicone oil removal

Before silicone oil removal all eyes had a reattached and stable retina. Postoperatively and at the last follow-up, out of 82 eyes, the retina remained attached in 59 eyes (72%); recurrent RD occurred in 23 eyes (28%) [Table 1]. Re-detachment occurred in 3 (10.3%) out of 29 eyes filled with 1000cs silicone oil, however 20 (37.7%) out of 53 eyes filled with 5000cs silicone oil developed re-detachment, this difference is statistically significant (P = 0.008). There was no statistically significant difference among various surgical indications in terms of re-detachment, however the overall re-detachment rate for eyes with RD and PVR was higher (34%). In this category, the re-detachment rate in eyes filled with 5000cs silicone oil and 1000cs silicone oil was 45% and 8.3% respectively (P = 0.0002). After silicone oil removal, the chance of having an attached retina was higher for eyes filled with 1000cs silicone oil than those filled with 5000cs silicone oil (relative risk = 5.25).

### Visual outcome following silicone oil removal

Overall, 28 eyes (34.2%) had improvement of visual acuity following silicone oil removal. Of these, 16 eyes belonged to the 1000cs silicone oil group [16/29 (55%)] and 12 eyes belonged to the 5000cs silicone oil group [12/53 (22.6%)]. Preoperative visual acuity of counting finger or less was seen in 65% of cases with 1000cs silicone oil and 73% of cases with 5000cs silicone oil. After silicone oil removal, visual acuity of counting fingers or less was observed in 45% and 77% respectively. Table 3 details patients' preoperative and postoperative visual acuity.

**Table 3:** Visual outcome before and after silicone oil removal.

Visual acuity	Before oil removal				After oil removal			
	
	Silicone 1000		Silicone 5000		Silicone 1000		Silicone 5000	
	No	%	No	%	No	%	No	%
	
LP*	6	20.7	19	35.9	5	17.2	27	50.9
FC†	13	44.9	20	37.7	8	27.6	14	26.4
5/20–6/60	8	27.6	13	24.5	12	44.4	7	13.2
6/45–6/15	2	6.9	1	1.9	4	13.8	5	9.4
Total	29	100	53	100	29	100	53	100

*LP = Light perception – †FC = Finger counting

Visual acuity of 6/120 or less was used as an outcome in a multivariate analysis and the association of factors such as initial visual acuity, intraocular pressure, type of oil viscosity, anatomic status of retina, age and the surgical indication were evaluated, while controlling for potential confounders. This analysis showed that only two factors were associated with visual acuity of 6/120 or less: initial visual acuity and type of silicone oil viscosity. Patients with initial visual acuity of 6/120 or less had a higher chance of having final visual acuity of 6/120 or less (OR = 32.2, 95%CI 7.4–140.2). Patients who had retained 5000cs silicone oil also had a higher chance of final visual acuity of 6/120 or less (OR = 7.9, 95%CI 1.9–32.2). Visual acuity varied to some extent with the basic underlying disorder that dictated silicone oil injection, however, these differences were not statistically significant.

### Intraocular pressure following silicone oil removal (IOP)

Overall, prior to silicone oil removal 43 of 82 eyes (52.4%) had elevated IOP (i.e. ≥21mmHg). After silicone oil removal, IOP remained high in only 8 eyes (9.8%), in 69 eyes (84.1%) it was within the normal range (21 mmHg > IOP > 5 mmHg) and 5 eyes (6%) had severe hypotony (≤ 4 mmHg) [Table 4]. There was no significant difference among the 4 categories of underlying disorders in regard to IOP, both before and after silicone oil removal.

**Table 4:** Intraocular pressure before and after silicone oil removal

IOP*	Before oil removal				After oil removal			
	Silicone 1000		Silicone 5000		Silicone 1000		Silicone 5000	
	No	%	No	%	No	%	No	%
<21 mmHg	14	48.3	25	47.2	26	89.7	43	81.2
>21 mmHg	15	51.79	28	52.8	2	6.9	6	11.3
Phthisical	-	-	-	-	1	3.4	4	7.5
Total	29	100	53	100	29	100	53	100

*IOP = Intraocular pressure

After oil removal, elevated IOP was present in 2 eyes (6.9%) in the 1000cs silicone oil group and 6 eyes (11.3%) in the 5000cs silicone oil group. Excluding eyes that developed retinal re-detachment following silicone oil removal, only 8 out of 59 eyes (13.5%) had elevated IOP of more than 21 mmHg, 50 eyes (84.7%) had IOP within normal range and only one eye (1.6%) was phthisical (≤4 mmHg).

Postoperative IOP ≥ 21 mmHg was used as an outcome in a regression model and association of factors such as initial visual acuity, preoperative IOP > 21 mmHg, type of silicone oil viscosity, anatomic status of the retina, age and type of underlying disorder were evaluated. This analysis showed that only the anatomic status of the retina had significant association with IOP and expectedly, eyes with re-detachment following silicone oil removal had an average of 8.97 mmHg lower IOP than those with an attached retina.

## Discussion

Since the invention of the vitrectomy instrument, the role of silicone oil as a vitreous substitute and retinal tamponade has expanded. More recently, the beneficial effects of silicone oil have been re-confirmed in a multicenter clinical trial by the silicone oil study group[7,8]. Even though silicone oil has proved to be a very useful tool and adjunct in the treatment of complicated retinal detachments, the question of the preferred silicone oil viscosity for use in clinical settings still remains unanswered.

Earlier studies have shown no difference in the tamponading force among purified silicone oils of various viscosities[1,3]. The most frequently used silicone oils are highly purified polydimethylsiloxanes with viscosities as low as 100cs to a maximum of 12500cs, however 1000cs and 5000cs define the viscosity range of currently used silicone oils in most vitreoretinal surgeries.

Low viscosity silicone oils are preferred by some surgeons because of easier surgical handling and removal from the vitreous cavity[2,5,6]. On the other hand, higher viscosity silicone oils are subject to decreased and delayed emulsification, so that the tamponading force lasts longer, which may provide better tamponade for some complex forms of retinal detachment that need a longer effect[2]. However, silicone oils of various viscosities have similar tamponading effects as long as emulsification of the oil has not occurred[3].

Despite these facts and the relative agreement on indications of silicone oil use and removal, the issue of preferred viscosity and time for removal deserve further investigation[9,10].

In this non-randomized study we evaluated the outcomes of successful complex retinal detachment surgery following removing silicone oils of two different viscosities, 1000cs and 5000cs. Prior to oil removal all eyes had a stable attached retina. In our patients, silicone oil emulsification occurred in all eyes to some degree. We may thus conclude that the tamponading force of silicone oil had been lost before removal. However, with this fact in mind, we observed an overall redetachment rate of 28%, which is almost similar to some other published reports on silicone oil removal before emulsification [10,11,16]. The majority of the recurrent retinal detachments occurred within 3 months of oil removal. Re-detachment rates in eyes with RD associated with PVR slightly exceeded other indications and interestingly the re-detachment rate in the 5000cs silicone oil group was significantly higher than the 1000cs group. The cause of this re-detachment following silicone oil removal was mostly residual traction and redevelopment of proliferative vitreoretinopathy that had led to reopening of preexisting retinal breaks, or formation of new retinal breaks as a result of surgical manipulations. These findings suggest that in making the decision to remove silicone oil from the eye, not only retinal stability, but also other factors that may aggravate PVR formation should be considered. In this study the re-detachment rate was higher for eyes filled with 5000cs silicone oil, especially eyes with RD and PVR. One possible explanation for this observation could be the use of 5000cs oil in more complex cases. Another explanation may be the length of time since primary vitrectomy surgery unrelated to the properties of the oil. It is possible that all retinas had an increased tendency to re-detach with extended follow-up and eyes that had been filled with 5000cs oil were seen later in the disease process due to longer retention period of the oil, therefore increasing the detection of re-detachment.

Overall, 34.2% of the eyes in our study experienced improvement of visual acuity following silicone oil removal. Elimination of the variability in refraction induced by the anterior curve of the silicone oil bubble as well as light diffraction induced by droplets of emulsified oil may have rendered the eye more amenable to optical correction.

Our data suggest that visual acuity prior to silicone oil removal and viscosity of silicone oil are both associated with final visual acuity: eyes with preoperative visual acuity of 6/120 or less and those filled with 5000cs silicone oil had less chance of obtaining a final visual acuity ≥ 6/120. There is no bias involved in the selection between the two varieties of silicone oil and that the selection was purely based on the availability of the same at the given point of time. Aside from the above-mentioned presumed selection bias, the longer duration of 5000cs silicone oil retainment (mean, 23.9 months) compared to 1000cs silicone oil (mean, 10 months) with its attendant pressure effect on the retina may somehow cause retinal damage. The other possible mechanism includes subclinical emulsification with gradual penetration and migration of oil droplets into the retina causing damage or toxicity and diminishing the of chance obtaining good visual outcome [17-20]. The difference between the retention time of 1000cs and 5000cs oil groups was due to earlier emulsification of silicone oil 1000cs and development of complications such as increased IOP and keratopathy. Indications for silicone removal in this study were the early detection of these complications such as emulsification alone or associated with increased IOP or keratopathy prior to removal of oil. This policy of postponing silicone oil removal until appearance of complications could skew our results toward a poorer outcome.

When elevated IOP (≥ 21 mmHg) following silicone oil removal was used as an outcome, in eyes with re-attached retina and controlling for potential confounders none of the factors in the multivariate regression analysis showed significant association with IOP. This may indicate that once silicone oil emulsifies, there should be no difference between the two different viscosities regarding IOP elevation.

## Conclusion

Accomplishing a stable, re-attached retina is the final goal of all retinal surgeons. The possibility of achieving this goal could be increased with the use of higher viscosity silicone oil for longer periods especially in complex forms of retinal detachment. However, based on our present experience we observed a poorer anatomic and functional outcome with the use of higher viscosity silicone oil (5000cs) as compared to lower viscosity silicone oil (1000cs). It should be emphesized that due to the limitations of retrospective studies the validity of all these observations should be reconfirmed by a randomized clinical trial.

## Abbreviations

*RD = Retinal detachment – †PDR = Proliferative diabetic retinopathy ‡IOFB = Intraocular foreign body – **PVR = Proliferative vitreoretinopathy.

## Competing interests

The author(s) declare that they have no competing interests.

## Authors' contributions

MS is the main researcher and the corresponding author. MMa has scheduled the cases and has recorded the data of the patients praoperative and follow-up sheets. MMo has revised the article and prepared the manuscript to be in the scientific format for publication. BR has helped in collecting the data and in statistics procedures.

## Pre-publication history

The pre-publication history for this paper can be accessed here:


